# A patient with medication-resistant epilepsy featuring psychosensorial and psychotic symptoms presenting with significant functional improvement on psychotherapeutic treatment: a case report

**DOI:** 10.1186/1752-1947-7-259

**Published:** 2013-11-11

**Authors:** Luigi De Benedictis, Alexandre Dumais, Luc Nicole, Christine Grou, Alain D Lesage

**Affiliations:** 1 Centre de recherche de l’Institut Universitaire en Santé Mentale de Montréal, Université de Montréal, 7401, Rue Hochelaga, Montréal, Québec H1N 3M5, Canada; 2 Centre de recherche de l’Institut Universitaire en Santé Mentale de Montréal, Institut Philippe-Pinel de Montréal, Université de Montréal, Montreal, Québec, Canada; 3 Department of Psychiatry, Institut Universitaire en Santé Mentale de Montréal, Université de Montréal, Montreal, Québec, Canada; 4 Department of Psychology, Institut Universitaire en Santé Mentale de Montréal, Université de Montréal, Montreal, Québec, Canada; 5 Centre de recherche de l’Institut Universitaire en Santé Mentale de Montréal, Montreal, Québec, Canada; 6 Department of Psychiatry, Université de Montréal, Montreal, Québec, Canada; 7 Institut Universitaire en Santé Mentale de Montréal, Montreal, Québec, Canada

**Keywords:** Partial complex epilepsy with psychosensorial symptoms, Integrated psychological therapy (IPT), Psychosis, Medication, Rehabilitation

## Abstract

**Background:**

Partial complex epilepsy with psychosensorial and psychotic symptoms remains a relatively rare condition that can sometimes be mistaken for an axis I psychiatric disorder. There is no specific treatment for this particular type of epilepsy, anti-epileptic medication being the cornerstone of therapeutic intervention with the occasional addition of neuroleptics. Lack of response to anti-epileptic agents is often a sign of poor prognosis and requires risky and sometimes invasive interventions with high morbidity for patients.

**Case presentation:**

We report the case of a 21-year-old right-handed Caucasian man of French-Canadian descent who was living with his mother immediately before being hospitalized in a psychiatric setting for the first time. He seemed obsessed with developing new concepts to reach a more ‘perfect’ existence. He also claimed feeling odd sensations in his mind and in his body that could be linked to some sort of ‘evolutionary’ process resulting from spiritual uplift. He reported non-specific visual hallucinations and what sounded like auditory hallucinations and telepathic powers. The first diagnosis was a possible schizophreniform disorder and our patient was hospitalized. Shortly afterwards, an electroencephalogram showed an important subcortical epileptic activity, compatible with partial complex epilepsy with psychosensorial and psychotic symptoms. Despite a negative response to medication, symptoms proper to this type of epilepsy were substantially alleviated using a psychotherapeutical treatment intended for patients with psychotic disorders, namely integrated psychological therapy (IPT). Significant functional improvement in our patient has been achieved since then.

**Conclusions:**

This case report illustrates that despite a negative response to medication, symptoms proper to this type of epilepsy could be substantially alleviated using psychotherapeutical treatment modalities. To the best of our knowledge, this is the first time such a finding has been reported in the scientific literature. This could open the way for new research themes and therapeutic interventions for such patients.

## Background

The possibility of a link between epilepsy and psychosis was considered scientifically plausible as early as the middle of the nineteenth century [1]. Interest in researching this subject increased in 1963, after Slater and Beard [2] concluded that the prevalence of schizophrenia was higher among patients with epilepsy than in the general population. Today, inter-ictal psychoses are considered to occur in about 7 percent of all patients with epilepsy and have been described in both partial and generalized epileptic syndromes [3]. The interval between seizure onset and psychosis is about 14 years [4], and some risk factors have been identified; they include female gender, familial aggregation of schizophreniform disorder and normal pre-morbid personality [5,6].

The precise neuropsychological process that causes patients with epilepsy to develop psychotic symptoms is still unknown [7]. However, it has been argued that the temporal lobes of the brain seem to be particularly relevant in explaining the phenomenon because of the fact that they bind complex perceptual inputs (auditory, olfactory and visual) to emotions [8]. Right temporal foci leads to external emotive or behavioral manifestations such as anger, sadness, elation, circumstantiality, viscosity and hypermoralism. By contrast, left hemisphere foci causes ruminative, intellectual tendencies such as religiosity, philosophical interest and a sense of personal destiny [4]. While some studies suggest that there is a link between schizophrenia and temporal lobe epilepsy and psychosis [9], others show they are completely different entities from a clinical and neurobiological aspect [10]. Refractory patients with epilepsy also have higher incidents of personality disorders, especially from cluster C (avoidant and dependent) [11], and inter-ictal dysphoric disorder, with prominent psychotic symptoms occurring almost exclusively among patients with the latter [12].

While several studies have shown the possible outcome of inter-ictal psychosis, to the best of our knowledge none have tried to determine the impact of psychotherapeutic treatment. Here, we present a report concerning a patient with pharmacologically resistant epilepsy, treated using psychological therapy usually used with patients experiencing non-organic psychotic disorders.

## Case presentation

Our patient was 21 years old when he was first hospitalized. This single, Caucasian, right-handed man was living with his mother and had finished high school five years earlier. During this period, he did not pursue any goal-oriented projects; he did not work or try to go back to school, and spent most of his time withdrawn in his bedroom. He had no siblings and very little contact with his father. He had been drug free for the past year and his family history for neurological and psychotic disorders was negative.

He was admitted to the emergency ward of a psychiatric hospital about four years ago after a court order was issued to evaluate his mental state. Three days earlier, our patient had been arrested after his mother called the police to report being assaulted and threatened by him. The fight took place after she had scolded him for being lazy and aloof. His initial psychiatric evaluation revealed a very fragile mental state for at least the five previous years. After quitting school because of a lack of motivation, our patient started to spend most of his time reading about subjects related to spirituality. Previously, he had been a relatively good student, with no disruptive behavior and above average marks. He seemed obsessed with developing new concepts to reach a more ‘perfect’ existence and thought that studying anything but these subjects was simply futile. The initial mental examination, performed at the time of his admission, revealed coherent speech but a perplexed attitude and what sounded like loose associations. He seemed suspicious, but he had no suicidal or homicidal thoughts. He claimed feeling odd sensations in his mind and in his body that could be linked to some sort of ‘evolutionary’ process resulting from spiritual uplift. He also reported non-specific visual hallucinations (seeing odd shapes and colored lines) and what sounded like auditory hallucinations and telepathic powers. There was no hypergraphism and hypermoralism. His medical history was not contributory. The initial diagnosis was a possible schizophreniform disorder and our patient was admitted. His Global Assessment of Functioning (GAF) scale score was marked at 25, and a dose of risperidone 1mg at bedtime was initiated.

An electroencephalogram (EEG) was performed shortly after his admission and revealed a background symmetric alpha rhythm of about 10Hz, predominantly in the posterior and temporal regions of the brain, coupled with an important subcortical epileptic activity with 3 to 5Hz of slow, pointed waves outbursts. The results of a thorough neurological examination were perfectly normal. However, our patient reported having frequent ‘déjà vu’ sensations and depersonalization tantrums over the past few years to the consultant neurologist. The neurologist concluded our patient was a case of partial complex epilepsy with psychosensorial and psychotic symptoms and prescribed levetiracetam 500mg twice a day. Meanwhile, the risperidone was ceased since our patient showed worsening of certain symptoms. A neuropsychological evaluation showed important deficits in executive functions (especially working memory and concentration) and lack of motivation, probably secondary to intellectual understimulation for a long period of time. However, since our patient had no developmental delay up to the middle of high school, his intellectual potential was probably superior and was enhanced with proper management and stimulation. A control EEG performed one month later showed no improvement and carbamazepine continued release was progressively increased to 400mg twice a day (blood concentration stabilized at 40μmol/L). The results of a computed tomography (CT) scan and cerebral magnetic resonance imaging (MRI) study were both normal. Follow-up EEGs (performed six months after his first admission) continued to show the same epileptic activity and carbamazepine was stopped and replaced by valproic acid (up to 500mg twice a day with blood concentration stabilized at 452μmol/L). In spite of this, his EEG results remained practically unchanged.

Even though abnormal epileptic activity remained, our patient showed significant improvement during this period. Three months after admission, our patient left the hospital and started an integrated psychological therapy (IPT) group three times a week, a program that combines neurocognitive and social cognitive interventions with social skills approaches for patients who are schizophrenic. IPT has been shown to be an effective rehabilitation approach for patients experiencing psychotic disorders [13]. In addition, he was also integrated into a rehabilitation home in order to practice these skills in a protected and proactive setting adapted to his strengths and weaknesses and willing to follow his pace of learning. Monthly individual cognitive-behavioral therapy (CBT) sessions, adapted for psychotic patients [14,15], were also initiated by the treating psychiatrist to further potentiate the treatment. In a matter of months, our patient realized his deficits in social interactions. When confronted with them, he tried to isolate himself by going back to his old thoughts on spirituality and pursuit of a perfect world. However, these beliefs were found to be primitive psychological defense mechanisms and our patient himself knew this. The previous visual and tactile hallucinations became much more scattered and were more the fruits of odd interpretations of reality. Almost a year and a half after being hospitalized, he started a job reinsertion program and is looking forward to moving into his own apartment. The final psychiatric diagnosis was a psychotic disorder and personality change due to a general medical condition (epilepsy). However, despite active epilepsy, non-responsive to medication, he has managed to control a lot of symptoms deriving from the intertwining of his organic and psychiatric disorders. A control neuropsychological evaluation performed more than a year after the first one revealed steady scores for executive functions, but significant improvement in speed of information processing. His most recent GAF scale score was 65.

## Discussion

In summary, significant functional improvement was achieved for severe psychotic symptomatology (delusions, social withdrawal and hallucinations) in our patient experiencing partial complex epilepsy using therapeutic methods that have proven to be effective in treating schizophrenic patients. To the best of our knowledge, this is the first case of a psychotherapeutic treatment used effectively in treating refractory epilepsy of this type [16].

When considering treatment options, one must keep in mind the forced normalization phenomenon in epilepsy, whereby improved seizure control is associated with a normalization of the EEG and the emergence of psychotic features [17] and dysphoria [12]. It is thought that abnormal synaptic activity may induce plastic changes through kindling mechanisms, and impair the naturally occurring homeostatic seizure-suppressing mechanisms (inhibitory factors) that maintain the inter-ictal state, with adverse consequence that these factors become dysfunctional, causing these symptoms when the epileptic activity is gone [7]. Therefore, it is not surprising to find contradictory results when outcomes of temporal lobe surgery in treating patients with epilepsy and concomitant psychotic and dysphoric symptoms are compared [18-20]. With regard to non-anti-convulsant medication, patients with epilepsy with inter-ictal psychosis seem to achieve higher remission rate with lower doses of anti-psychotic drugs compared to patients with schizophrenia [21]. With regard to dysphoric symptoms, they usually respond well to anti-depressant medication [12]. However, given the pro-convulsant properties of neuroleptic drugs in general, they should be prescribed with a great deal of parsimony when EEGs remain abnormal [22]. Reports of successful treatment of psychosis associated to complex partial seizures have shown good response to anti-convulsant medication such as carbamazepine and valproic acid [23], but this was not the case for our patient, which further underscores the possibility that the psychotherapeutic component of the treatment plan explains, at least to some extent, the progress noted for our patient. Finally, anti-epileptic drugs have been shown to decrease cognitive functioning [24], which was not the case with our patient; he remained stable and even improved on some fronts (for example, speed of information processing).

## Conclusions

It seems possible that both partial complex epilepsy with psychotic symptoms and chronic psychotic disorders (such as schizophrenia) share more etiologic similarities than had been thought until now. Comprehensive psychological treatment has been proved not only to alleviate severe psychotic symptoms and cognitive deficits in schizophrenia [25-27], but also to modify cerebral functioning [28]. Considering that anti-convulsant medication may be ineffective and that other neuroleptic drug and brain surgery can be risky, neurocognitive and social cognitive psychotherapeutic interventions may be an interesting treatment modality in the future for patients with epilepsy with psychosensorial and psychotic symptoms, as for other organic disorders with psychiatric symptoms [29].

## Consent

Written informed consent was obtained from the patient for publication of this case report and any accompanying figures. A copy of the written consent is available for review by the Editor-in-Chief of this journal.

## Competing interests

The authors declare that they have no competing interests.

## Authors’ contributions

LDB and AD were involved in the initial writing of the manuscript. SH provided major editing changes. LN and LDB were primarily involved in the care of our patient. CG carried out the different neuropsychological assessments. ADL provided intellectual contributions to the content of the manuscript as well as editorial assistance. All authors have read and approved the final version of the manuscript.
